# Fabrication of Electrospun Polylactic Acid/Cinnamaldehyde/β-Cyclodextrin Fibers as an Antimicrobial Wound Dressing

**DOI:** 10.3390/polym9100464

**Published:** 2017-09-21

**Authors:** Yaowen Liu, Xue Liang, Rong Zhang, Wenting Lan, Wen Qin

**Affiliations:** 1 College of Food Science, Sichuan Agricultural University, Yaan 625014, China; xue6liang17@163.com (X.L.); 18227593325@163.com (R.Z.); 18227593253@163.com (W.L.); qinwen@sicau.edu.cn (W.Q.); 2 School of Materials Science and Engineering, Southwest Jiaotong University, Chengdu 610031, China

**Keywords:** electrospinning, polylactic acid (PLA), cinnamaldehyde, β-cyclodextrins

## Abstract

Cinnamaldehyde (CA) was successfully encapsulated in β-cyclodextrin (β-CD), and polylactic acid (PLA)-based composite fibers were prepared by incorporating CA/β-CD via electrospinning. Morphological, structural, spectral, and antibacterial properties of different weight ratios of PLA:β-CD/CA (88:12, 94:6, 97:3, and 98.5:1.5) and PLA/CA/β-CD fibers were investigated. PLA and CA/β-CD were incorporated by mixing of CA/β-CD inclusions to enhance the viscosity of the mixed solution. The mechanical properties and hydrophilicity of nanofibers were improved following the addition of CA/β-CD. Moreover, CA/β-CD improved the antibacterial activities of the mixture against *Escherichia coli* and *Staphylococcus aureus*. PLA/CA/β-CD-3 exhibited excellent antibacterial effects and low cytotoxicity. Thus, our study showed that PLA/CA/β-CD fibers may have applications as wound dressing materials and for use in other biomedical applications.

## 1. Introduction

Cinnamaldehyde (CA) is a botanical essential oil derived from cinnamon bark. Due to its excellent antibacterial effects, low toxicity, and strong antioxidant properties, CA is widely used in the food, pharmaceutical, biomedical, and cosmetics industries [1]. However, CA is highly volatile in nature, unstable, and readily undergoes oxidation upon exposure to oxygen, light, and heat. In recent years, numerous studies have demonstrated the significance of polymer blends, such as polylactic acid (PLA), poly(ethylene oxide) (PEO), polycaprolactone, poly(vinyl alcohol) (PVA), and polyethylene terephthalate, in maintaining the stability and bioactivity of CA [2]. CA-containing polymers can form films, gels, beads, and particles. Notably, in vitro experiments have shown that addition of CA to composite films applied to slices of bread and cheese during storage effectively inhibited *Penicillium* and *Aspergillus niger* growth, thereby prolonging the storage period of bread and cheese [3]. Additionally, using Schiff alkali chitosan as carrier materials, researchers prepared a loaded composite of CA and graphene and found that this composite inhibited *Rhizopus* growth on sliced bread [4]. Copolymer fibers prepared by electrospinning as a biodegradable multilayer structure of poly butadiene copolymer acid, corn protein, and CA poly-3-hydroxybutyrate showed antibacterial effects, suggesting that this polymer could have applications in the establishment of an active bio-based multilayer system for food packaging applications [5]. Moreover, a cinnamon essential oil/cyclodextrin inclusion complex (CEO/b-CD-IC) integrated into PLA nanofibers was prepared via electrospinning and co-precipitation, showing better antimicrobial activity than the PLA/CEO nanofilm, effectively prolonging the shelf life of pork and supporting its potential applications in active food packaging [6]. Further studies have shown that electrospun PVA/CEO/β-cyclodextrin (β-CD) antimicrobial nanofibrous films (average diameter of 240 ± 40 nm), fabricated under optimal conditions with a mild electrospinning process to generate nanofibrous mats, showed higher antimicrobial activity than PVA/CEO/β-CD films. Additionally, these PVA/CEO/β-CD nanofibrous mats could prolong the shelf-life of strawberries, indicating their potential applications in active food packaging [7].

The controlled release of essential oils from food packaging structures is mainly based on concentration-dependent passive diffusion. Essential oils are known to have anticancer [8], antifungal [9], antioxidant [10], anti-inflammatory [11], and antimutagenic effects [12], highlighting their utility as matrix materials for wound dressing applications. However, essential oils have poor physical properties, including hydrophobicity and susceptibility to degradation, hindering their use in tissue engineering applications. In order to overcome these disadvantages, essential oils have been encapsulated into films, gels, beads, and particles. Essential oils can be delivered via carrier-solutions, polymer derivatives, or encapsulation in solid particles/films [13,14]. Liakos et al. fabricated sodium alginate and essential oil composite films by the drop and cast method and found that films containing different percentages of essential oils could show antibacterial effects against *Escherichia coli* and *Candida albicans*. Such films could be used as disposable wound dressings, food packaging, or medical device protectants [15]. Many essential oils, such as thyme, lavender, peppermint, cinnamon, and lemongrass oils, have been found to exhibit specific antimicrobial properties [16]. For example, eugenol and limonene added to nanofluid-based magnetite have been shown to have antimicrobial properties in wounds, and *Eugenia carryophyllata* essential oil showed antifungal effects in biomedical applications after stabilization using an iron oxide/oleic acid core/shell nanostructure [17].

Electrospinning is a well-established technique to generate micro- to nanometer-scale polymeric fibers [18]. This method can also be used to encapsulate essential oils because it is responsive to changes in the surrounding atmosphere, allows retention of controlled release and consecutive delivery of multiple active ingredients, and creates highly porous and permeable scaffolds with high surface-to-volume ratios, which are ideal for wound dressings [19]. Rieger et al. obtained chitosan/CA/PEO electrospun fibers without surfactants. The release of CA from the chitosan/CA/PEO nanofiber mats directly influenced their cytotoxicity against *Pseudomonas aeruginosa*. The release of CA from the chitosan/PEO nanofiber mats demonstrated the potential of this material as a flexible scaffold that could alleviate nosocomial infections by delivering a broad-spectrum natural antimicrobial agent [20]. Later, researchers developed a new therapeutic wound dressing in which CA and hydrocinnamic alcohol were electrospun in chitosan (CS)/PEO nanofibers; these composite fibers broadened the delivery of therapeutics, allowing hydrophobic agents to be delivered by hydrophilic nanofiber mats. However, no studies have evaluated the encapsulation of CA using β-CD as a wound dressing [21]. 

Accordingly, in this study, we carry out CA encapsulation using β-CD and then incorporated CA/β-CD into PLA via electrospinning. We investigate the morphology, structure, release behavior, and antibacterial ability of PLA/CA/β-CD nanofibers. We hypothesized that the CA-loaded PLA/β-CD nanofibers would show low cytotoxicity, suggesting the potential use of these nanofibers in future biomedical and pharmaceutical applications.

## 2. Materials and Methods

### 2.1. Materials

PLA (*M*w = 15,000 g/mol) was synthesized in our laboratory, as previously reported [22]. CA (≥93%, FG, *M*w = 132.16 g/mol) was obtained from Sigma-Aldrich (St. Louis, MO, USA). β-CD, dichloromethane (DCM), and *N*,*N*-dimethylformamide (DMF) were supplied by Chengdu Institute of Organic Chemistry, Chinese Academy of Science (Chengdu, China). Dulbecco’s modified Eagle’s medium (DMEM) was obtained from Gibco BRL (Rockville, MD, USA), and bovine serum albumin was purchased from Sigma-Aldrich. Deionized water was prepared using a Millipore Milli-Q ultra-pure water system. The microorganism strains of *E. coli* (ATCC 29522) and *Staphylococcus aureus* (ATCC 25923) were maintained in our laboratory. Difco Luria-Bertani (LB) broth was procured from BD Biosciences Co. (Woburn, MA, USA). All chemical reagents used were of analytical grade or better, and were purchased from Changzheng Regents Company (Chengdu, China) unless otherwise indicated.

### 2.2. Preparation of β-CD/CA Particles

The inclusion complex of CA and β-CD was prepared using a co-precipitation method. Briefly, 10 g β-CD was dissolved in 100 mL distilled water and stirred using a magnetic stirrer (RT5; IKA, Staufen im Breisgau, Germany) at 40 °C for 2 h. One milliliter of CA was then slowly added to the β-CD solution with stirring at 700 rpm for 90 min to obtain a weight ratio of CA: β-CD of 10:90. The solution was cooled to room temperature, and the final solution was freeze-dried to collect β-CD/CA particles. Finally, the sample was washed twice with 30% ethanol solution and then freeze-dried into power for 24 h.

### 2.3. Preparation of PLA/β-CD/CA Fibers

PLA solution (15%, *w*/*w*) was prepared by dissolving 15 g PLA in a 100 mL co-solvent system of DCM to DMF with a ratio of 3:1 (*v/v*) with constant stirring for 3 h. The PLA/β-CD/CA fibers were then fabricated by adding a certain amount of CA/β-CD into the PLA solution. The polymer solution was added to a 5 mL syringe attached with a blunt metal needle as the nozzle. The distance between the collector and needle was about 15 cm, and the flow rate was set 1.0 mL/h by a precision pump (Zhejiang University Medical Instrument, Hangzhou, China). The electrospinning voltage was controlled within 20 kV through a high-voltage statitron (Tianjing High Voltage Power Supply Company, Tianjing, China). A grounded plate-type collector was used to collect the fibers. Different fibers were vacuum-dried at room temperature overnight to remove any solvent residue prior to further use. The electrospun fibers were denoted as PLA/β-CD/CA-12, PLA/β-CD/CA-6, PLA/β-CD/CA-3, and PLA/β-CD/CA-1.5 for weight ratios of PLA:β-CD/CA of 88:12, 94:6, 97:3, and 98.5:1.5, respectively.

### 2.4. Characterization of PLA/β-CD/CA Fibers

The viscosity of the solution was read directly from a Brookfield viscometer (Model DV-II + Pro, Middleboro, MA, USA). The tests were carried out in triplicate, and the data were presented as average values. The morphology of fibers was investigated by a scanning electron microscope (SEM, FEI Quanta 200, Eindhoven, The Netherlands) equipped with a field-emission gun (20 kV) and a Robinson detector after 2 min of gold coating to minimize the charge effect. Attenuated total reflectance Fourier-transform infrared (ATR-FTIR) spectrometry was used to identify the chemical structures of PLA/β-CD/CA fibers and the possible interactions between their components. A small section cut from each composite fiber was used. The samples were analyzed with a resolution of 4 cm^−1^, aperture setting of 6 mm, scanner velocity of 2.2 kHz, sample scan time of 32 s, and 100 total scans per sample, in the range of 500 to 4000 cm^−1^. Spectral outputs were recorded in the absorbance mode, as a function of wave number, using a Bruker 66 spectrometer (Karlsruhe, Germany) [23]. The diameters of fibers were determined manually from SEM images using Image J software, and the specific methodology was referred to http://rsbweb.nih.gov/ij/plugins/index.html. The analysis of the fiber diameter was evaluated at three randomly-selected SEM images with a magnification of 5000×, and at least 50 different sites from each image were randomly chosen and measured to generate an average value as previously reported [24]. To measure the mechanical properties, fibers with dimensions of 5 mm × 50 mm were placed between the jaws of a mechanical testing machine (Instron 5567, Canton, MA, USA). A constant elongation (3 mm·min^−1^) was applied along the longitudinal axis of the vessel until rupture, to obtain a stress-strain curve, and the ultimate strength and strain at failure were obtained as described previously [25]. The conductivity (*σ*, S/cm) was measured using the four-point probe technique, the fibers were punched into small strips (10 × 40 mm^2^). Then one pair of probes was used for the current injection while the other pair was used for the voltage measurement [20], and calculated based on the equation:

*σ* = *l*/(*SRs*)

where *l* is the distance between reference electrodes, *S* is the cross-sectional area of the fibrous sample, and *Rs* is the ohmic resistance of bulk samples. *Rs* was measured with an impedance/gain-phase analyzer (Solartron 1260, Farnborough Hampshire, UK) and an electrochemical interface (Solartron 1287, Farnborough Hampshire, UK), as described previously [24]. A video-based optical contact angle meter (Data Physics OCA 15EC, Filderstadt, Germany) was used to measure the hydrophilicity of the fibers. Briefly, 4 cm × 4 cm samples were fixed on a glass microscope slide, placed on the meter stage, and instilled with a 5 µL drop of water. At least five contact angles at different locations were measured, and the average contact angle for each sample was obtained [25]. The release behavior of CA from the PLA/β-CD/CA fibers was assessed using the dialysis method as described previously [23]. Briefly, the fibers were punched into squares with a length of 1 cm, and then soaked to a dialysis bag, containing 50 mL of phosphate buffered saline and 20% ethanol. The dialysis bag was adjusted to pH 7.4 at 35 °C, which simulated the human body surface environment. All samples were incubated under gentle agitation and collected every 10 h for 60 h. The percentage of released CA was measured by a UV–Vis spectrophotometer at 275 nm [23]. Release studies were analyzed using the parameters in Peppas’ equation [26]:

*Q_t_* = *A* × *t^n^*

where *Q_t_* is the cumulative percent of CA released at time *t*, *A* is a constant incorporating geometric and structural features of the nanoparticles, and *n* is the release exponent that indicates the release rate mechanism. Values of *n* less than 0.43 indicate that the dominant release mechanism is Fickian diffusion (case I transport); values between 0.43 and 0.85 indicate a non-Fickian diffusion mechanism, and values greater than 0.85 indicate a Case II release mechanism.

### 2.5. Antibacterial Test

The antimicrobial activity of PLA/β-CD/CA fibers against *E. coli* and *S. aureus* was as previously described [20]. Briefly, the sample was punched into a rectangle measuring 10 mm × 20 mm, and then immersed into test tubes containing 0.5 mL of inoculum (approximately 10^7^ CFU/mL of tested bacteria). The test tubes were shaken at 200 rpm and 37 °C, and the absorbance at 600 nm was measured at selected time intervals. At each time point, 100 μL of the bacterial suspension was spread onto LB plates using the spread plate method, and the loss of bacterial activity was applied to measure the colony-forming units (CFU) of *E. coli* and *S. aureus* [27].

### 2.6. Cell Viability Assay

The viability of CCC-HSF-1 human skin fibroblasts (HSFs) on fibrous mats was using MTT assays. Briefly, HSFs were cultured in DMEM (Gibco BRL) supplemented with 10% heat inactivated fetal bovine serum (Gibco BRL). The cells were seeded on different fibers in 96-well plates at a density of 1 × 10^5^ cells/cm^2^ and incubated in an incubator with 5% CO_2_. After culturing for 24 h, the medium form each well was replaced with MTT solution and the plates incubated at 37 °C for 3 h. After removal of the MTT solution, acid isopropanol was added into each well and incubated for 1 h at room temperature according to the reagent instructions. One hundred fifty microliters of incubated medium was pipetted into a 96-well tissue culture plate, and the absorbance at 570 nm was measured for each well using a UV spectrophotometer. This experiment was repeated five times.

### 2.7. Statistical Analysis

The values are expressed as means ± standard deviations (SDs) and were analyzed by one-way analysis of variance followed by Tukey’s post-hoc test to discern the statistical differences between groups. Differences with *p* values of less than 0.05 were considered statistically significant.

## 3. Results and Disccusion

### 3.1. Topological Characterization of Fibrous Mats

**Figure 1:**
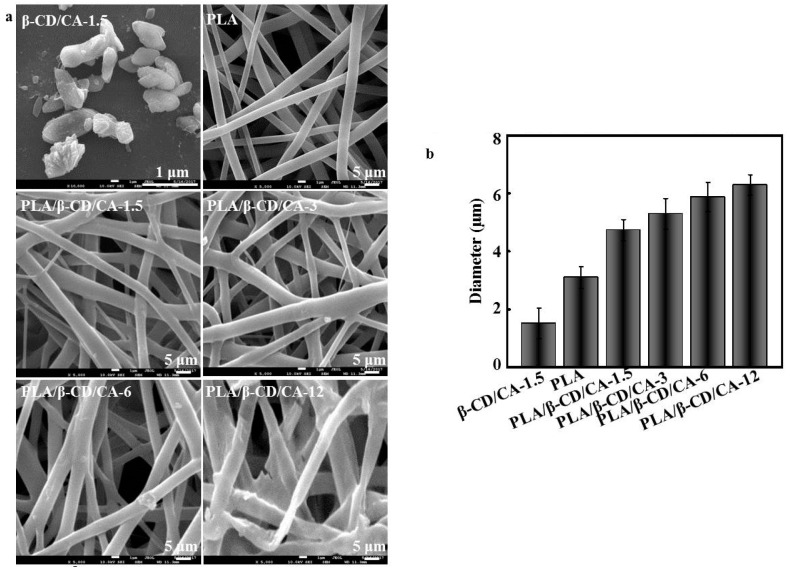
(**a**) SEM images of β-CD/CA-1.5 particle and PLA/β-CD/CA fibers; and (**b**) the average diameters of different fibers determined from SEM images.

Figure 1a summarizes the morphologies of PLA/β-CD/CA fibers, produced by electrospinning. The β-CD/CA particles appeared as multi-tier ellipse-shaped particle, and the small particles adhered to one another. A similar phenomenon was observed by Wen et al. [6]. The composites of PLA/β-CD/CA-1.5 to PLA/β-CD/CA-6 fibers showed a cylindrical structure and no visible separation of the particles from the fiber matrix (without beads), confirming that the β-CD/CA powder was successfully encapsulated into the PLA fibers. Notably, dispersed β-CD/CA-12 fibers could not be obtained, and bead defects were observed when β-CD/CA content was higher than 12%. The low viscosity of the polymer solution could result in an increase in the surface tension of the solvent, which favored the formation of beads [28].

The diameter of PLA/β-CD/CA fibers increased as the amount of β-CD/CA increased, and a relatively uniform fiber diameter distribution was observed (Figure 1b). Higher concentrations of β-CD/CA caused a gradual linear increase in the diameter of PLA/β-CD/CA fibers (PLA/β-CD/CA-1.5: 4.15 ± 0.36 μm; PLA/β-CD/CA-3: 4.94 ± 0.41 μm; PLA/β-CD/CA-6: 5.45 ± 0.52 μm; and PLA/β-CD/CA-12: 5.99 ± 0.64 μm). In addition, the average diameter of the PLA/β-CD/CA fibers was significantly higher than that of pure PLA fibers (3.17 ± 0.23 μm; *p* < 0.05).

### 3.2. FT-IR

**Figure 2:**
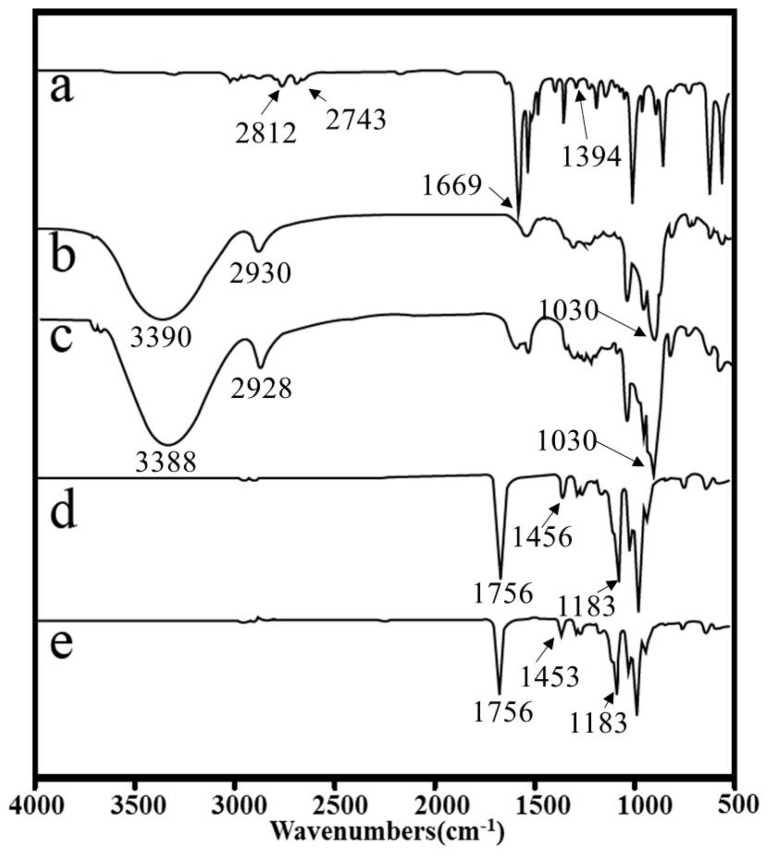
FT-IR spectra of (**a**) CA; (**b**) β-CD; (**c**) β-CD/CA; (**d**) pure PLA fibers; and (**e**) PLA/β-CD/CA-1.5 fibers.

Figure 2 shows FT-IR spectra of PLA, CA, β-CD, CA/β-CD, and PLA/β-CD/CA fibers. The characteristic absorption peaks of PLA were observed at around 1756, 1456, and 1183 cm^−1^ due to the –COOH, –CH_3_, and –OH stretching vibrations, respectively, as reported in the literature [29]. The FT-IR spectrum of CA showed characteristic peaks at 2812 and 2743 cm^−1^ due to the C–H stretching vibration; 1669 cm^−1^ represented the C=O stretching vibration, and 1394 cm^−1^ represented the C–H in-plane bending vibration in –CHO [23]. The characteristic absorption peaks of pure β-CD were observed at around 1030, 2930, and 3390 cm^−1^ due to the C–O–C, C–H, and O–H stretching vibrations, respectively [6]. From the spectrum of CA/β-CD, characteristic peaks of CA and β-CD were observed, confirming the presence of both CA and β-CD in the sample. However, the intensity of CA was markedly decreased because most peaks depended on the concentration of the encapsulated CA, similar to previously-published findings [30]. Weak peaks for β-CD and CA were found in the spectrum of PLA/β-CD/CA fibers, indicating that β-CD and CA were efficiently incorporated into the PLA. In addition, the characteristic peaks of CA at 1394 cm^−1^ and 1669 cm^−1^ shifted to 1453 cm^−1^ and 1756 cm^−1^ for PLA/β-CD/CA, respectively. Thus, there were many interactions between the β-CD/CA and PLA; a similar phenomenon was also observed in previous studies [6].

### 3.3. Physical Characteristics of Different Fibrous Mats

Table 1 summarizes the physical characteristics of the electrospun fibers at different concentrations of β-CD/CA. The PLA concentration had a significant effect on the viscosity of PLA/β-CD/CA; higher PLA concentrations resulted in greater viscosity (increased from 45.27 ± 3.54 to 129.15 ±12.61 mPa·s) because the β-CD/CA inclusion filled the void of PLA [31]. Fatma et al. suggested that this effect may be related to the presence of β-CD crystals in the PLA solution and/or interactions between the β-CD and PLA polymer chains [30]. The conductivity of fibers increased as the CA concentration increased from 0.037 ± 0.063 to 0.058 ± 0.079 μS/cm (*p* > 0.05); this could be attributed to the critical β-CD/CA concentrations, and higher affinities of the hydrophilic groups of β-CD for CA during particle formation [32]. Although the addition of β-CD/CA in PLA did not affect the formation of PLA/β-CD/CA fibers, it affected the mechanical properties of the fibers. Table 1 shows the mechanical properties of PLA nanofibers with different β-CD/CA contents. The addition of β-CD/CA l decreased both the stress and strain from PLA/β-CD/CA-1.5 to PLA/β-CD/CA-6 fibers slightly (*p* > 0.05), and the Young’s modulus was gradually increased from 64.21 ± 7.48 to 76.47 ± 8.27. We showed that PLA/β-CD/CA-12 fibers exhibit a significant decrease in elongation at break (*p* < 0.05), suggesting that when a large amount of inclusion was added; the inclusion filled small voids of PLA fibers, thereby leading to the formation of fibers and increasing the breakpoint. 

### 3.4. Surface Hydrophilicity of PLA/β-CD/CA Fibers

**Figure 3:**
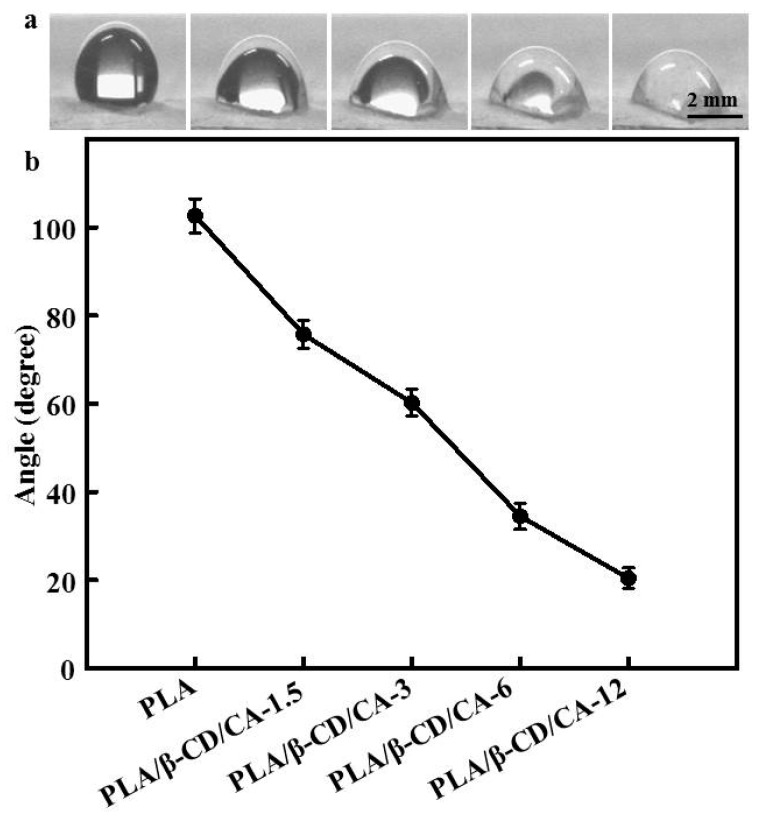
(**a**) Images of water contact angle of different fibers; and (**b**) average water contact angles of the fibers; *n* = 6.

Water contact angles of PLA/β-CD/CA fibers are shown in Figure 3. Pure PLA fibers are hydrophobic, and our results showed that addition of β-CD/CA could significantly decrease the water contact angle; water contact angles of PLA/β-CD/CA-1.5, PLA/β-CD/CA-3, PLA/β-CD/CA-6, and PLA/β-CD/CA-12 were 76.4° ± 5.1°, 62.4° ± 4.3°, 32.7° ± 2.1°, and 19.2° ± 1.2°, respectively. All water contact angles were less than 90, confirming the hydrophilic behavior of PLA/β-CD/CA fibers and the presence of hydrophilic β-CD/CA with abundant –OH groups on the surface of the composite fiber [6]. Indeed, incorporation of a higher concentration of β-CD/CA resulted in the formation of more hydrophilic PLA/β-CD/CA fibers.

### 3.5. Release Characteristics of PLA/β-CD/CA Fibers

**Figure 4:**
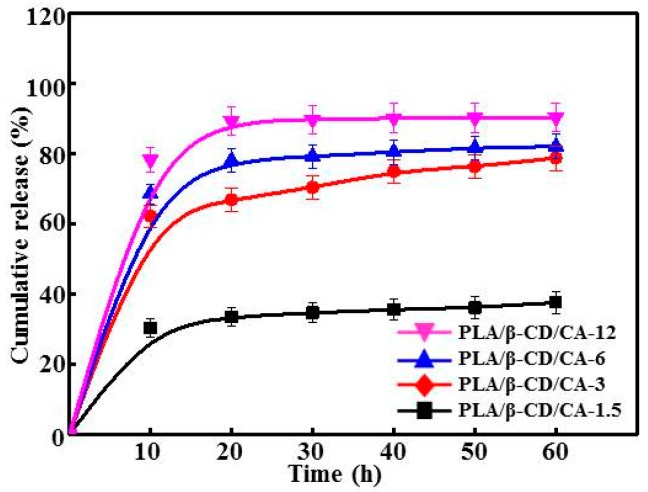
Antibacterial activity. The cumulative release profiles of CA from different fibers; *n* = 6.

The release profiles of CA from PLA/β-CD/CA fibers are shown in Figure 4. Each curve of the PLA/β-CD/CA fibers showed a slight initial burst within the first 15 h, due to the CA absorbed or loosely bound near the surface of the fibers. After 15 h, PLA/β-CD/CA-12 with sphere-fiber morphology resulted in uncontrolled release of CA. The release of CA from PLA/β-CD/CA-1.5, PLA/β-CD/CA-3, and PLA/β-CD/CA-6 exhibited a gradual accumulation. Moreover, the cumulative release percentage increased with increasing CA contents. In contrast, the cumulative amount of CA released from PLA/β-CD/CA-6 fibers was significantly higher than that from PLA/β-CD/CA-1.5 fibers, indicating that increased loading of CA correlated with increased release during the 20 h period. One possible reason is that the CA was wrapped in PLA/β-CD/CA-1.5 fibers and diffused through a longer distance than others. After 20 h, more than 60% CA was released from PLA/β-CD/CA-3 and CA showed a significantly gradual increase in the accumulative release compared with others (*p* < 0.05). This could be explained by the hypothesis that incorporation of CA may destroy the interaction between β-CD and PLA chains, which would have favored the excellent release percentage of CA. In addition, for the PLA/β-CD/CA-3 sample, we observed values of 0.43 < *n* < 0.85, which demonstrated that CA release was controlled by diffusion-swelling. This could be a result of the low degree of swelling and the presence of CA at the surface or in the exterior layer of β-CD [23]. Yet, the release behaviors of CA were varied considerably depending on the morphology and composition of PLA/β-CD/CA fibers [23]. 

**Figure 5:**
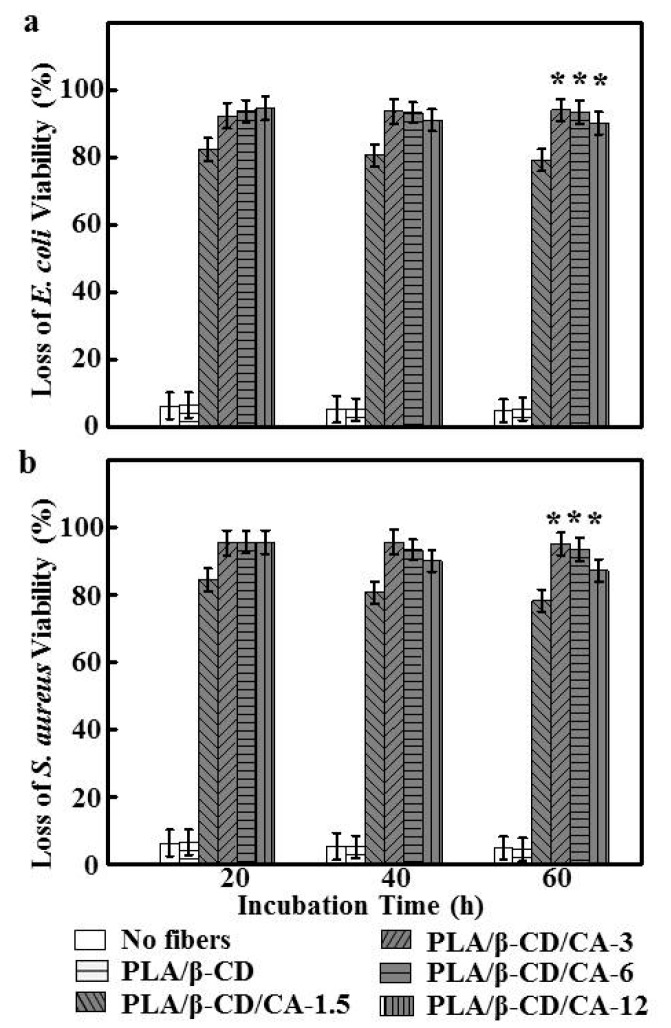
The loss of (**a**) *E. coli* and (**b**) *S. aureus* viability as a function of incubation time for different fibers (*n* = 6, *** means *p* < 0.05).

The mechanisms underlying the antibacterial activity of PLA/β-CD/CA fibers are not fully understood. Even though there are many reports proposing different mechanisms, there is no consensus [33,34]. Tiwari et al. have demonstrated that CA-loaded electrospun fibers can effectively inhibit the growth of both Gram-positive and Gram-negative bacteria, possibly due to CA leading to enzyme inactivation or protein denaturation [35]; thus, the antimicrobial activities of PLA/β-CD/CA fibers have been attributed to CA. As illustrated in Figure 5, PLA/β-CD fibers did not show any antibacterial activity, indicating that the PLA/β-CD matrices did not exert any antibacterial effects. PLA/β-CD/CA-1.5 fibers showed low antimicrobial activity against both *E. coli* (82.45% ± 2.56%) and *S. aureus* (79.83% ± 2.41%), relative to the release amount of CA from the fibers and bacteria exhibited sensitive concentration-dependent antibacterial effects. Except for the PLA/β-CD/CA-1.5 fiber, all PLA/β-CD/CA fibers completely inhibited the growth of *E. coli* and *S. aureus* for the first 20 h, which could result in the rapid release of CA adsorbed on the surface, allowing the CA concentration to reach the threshold for efficacy, consistent with a report by Hosseini et al. [36]. After 20 h, higher concentrations of β-CD/CA resulted in strong interactions between PLA and β-CD/CA and a slower reaction rate, thereby blocking additional release of CA and decreasing antibacterial efficiency. In contrast, for PLA/β-CD/CA-3 fibers, stable antibacterial activity (greater than 90%) was observed after 60 h. Further studies are needed to determine the release kinetics of CA from the blend fiber surface. Nguyen et al. also demonstrated that effective antibacterial materials should decrease the microbial concentration by at least 30%; PLA/β-CD/CA-3 fibers could satisfy this criterion, exhibiting excellent antibacterial efficiency [37]. Thus, these fibers could effectively prevent microorganism infection and were safe for humans, suggesting potential applications as a wound dressing. 

### 3.6. Viability of CCC-HSF-1

**Figure 6:**
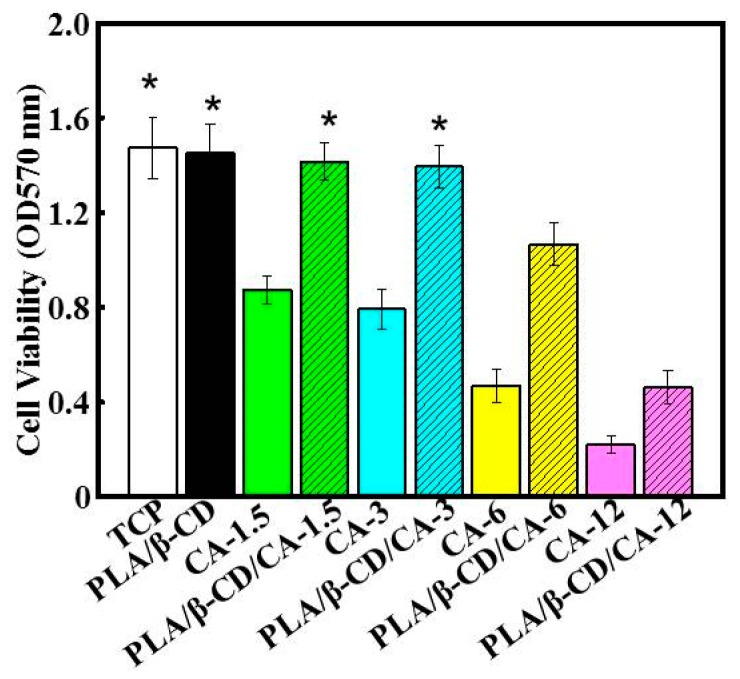
Cytotoxicity of different fibers on CCC-HSF-1 using MTT assay (*n* = 6, * means *p* < 0.05).

Figure 6 shows that PLA/β-CD/CA fibers could significantly improve cell viability compared with that of cells grown on CA alone (*p* < 0.05). This result could be explained by the encapsulation of various concentrations of CA into the PLA matrix. The cytocompatibility of polymer/CA composites has been demonstrated in numerous studies, despite the fact that CA was used at toxic concentrations [15]. Our results also revealed that concentration-dependent cell viability was observed following treatment with CA alone, or encapsulated CA. Cells exposed to CA alone showed toxic effects with activities of 52% and 74% for CA-1.5 and CA-6, respectively. These results could be attributed to the presence of various powerful anticancer components in CA, which were responsible for the reduced cell viability [25]. In contrast, PLA/β-CD/CA-1.5 and PLA/β-CD/CA-6 showed lower toxic effects, with decreases in viability of about 2% and 27%, respectively. Higher loading of CA is expected to increase toxic activity, consistent with the finding that PLA/β-CD/CA-12 showed the highest cytotoxicity of all examined PLA/β-CD/CA fibers (*p* < 0.05) [15]. In particular, PLA/β-CD/CA-3 showed less than 3% toxic activity compared with CA at the same concentration, demonstrating its suitability for wound dressing applications. These findings also confirmed that encapsulated CA could not directly reach individual cells to deliver CA [25]. 

## 4. Discussion

Electrospun fibers have received a great deal of attention in antimicrobial wound dressing, although the optimization of PLA/CA/β-CD fibers is rather limited. In the current study, CA-loaded electrospun fibers were fabricated with various fiber morphologies. Many factors, including solvents, polymers, voltages, and collecting distances, could influence the electrospun nanofibers. Thus, our results could be explained by the different conductivities and viscosities of the solutions, consistent with previous studies. For example, Liu et al. demonstrated that an increase in the concentration of polymer solution resulted in increases in viscosity and mean fiber diameter [23], and Saquing et al. showed that incorporation of β-CD/CA into a PLA solution decreased the conductivity of electrospinning suspensions, thereby decreasing the surface charge density of the spinning jet and increasing the diameter of PLA/β-CD/CA fibers [38]. The viscosity and conductivity of the polymer solution were consistent with SEM images. When the PLA concentration was over 12%, the viscosity was too low to form continuous nanofibers, and the lower conductivity resulted in the accumulation of beads on the nanofibrous film, forming an unstable Taylor cone at the needle tip. Bhardwaj et al. showed that lower conductivity would result in less stretching of the jet, thus producing nanofibers with larger diameters [39]. These findings suggested that β-CD was well suited for encapsulation of CA, consistent with the results of previous studies [40]. 

The increase in CA concentration decreased the mechanical properties of PLA/CA/β-CD fibers. First, the β-CD/CA acted as filler and dispersant in PLA/CA/β-CD fibers. Second, the degree of decrease in mechanical properties of the fibers was strongly dependent on the concentration and molecular structure of β-CD/CA; similar results have been found using β-CD in PLA nanofibers [30]. Moreover, Ioannis et al. proved that encapsulation of essential oils into plasticized and surfactant laden films only slightly decreased the Young’s modulus of the films, and the percent elongation at break of the films was reduced gradually as essential oils were added [15]. Moreover, increased CA concentrations resulted in lower viscosity, forming fewer macromolecular entanglements that were looser and crisper [41]. As expected, the CA concentration in the solution decreased the surface hydrophobicity of PLA fibers, due to the high hydrophilicity of β-CD and CA (Figure 4). Figure 5 summarizes the rate of CA release after 60 h, these results showed a similar initial burst release period. Particularly for PLA/CA/β-CD-3, constant release was eventually observed. There may be two steps to CA release into the medium. First, water molecules diffuse into the PLA/CA/β-CD fibers, and CA is released abruptly from β-CD embedded in the surface of PLA/CA/β-CD fibers. CA will then react with PLA/β-CD, allowing internal CA release to occur, and CA release slows down. CA release is also influenced by the dissolution and swelling behavior of the matrix polymer [34]. 

In this study, the antibacterial properties of fibers were evaluated in *E. coli* and *S. aureus*. The concentration of CA and the formation of PLA/CA/β-CD fibers have also been proposed as reasons for the antibacterial activity, where the accumulation of CA in fibers cause cell permeability and the death of microorganisms. Although the exact mechanism responsible for the antibacterial activity is unclear, and may be multifactorial, it is clear that PLA/CA/β-CD-3 fibers result in higher antibacterial activity than PLA fiber, Moreover, CA in the matrix of PLA/CA/β-CD-3 fibers had significant effects on antibacterial activity, and CA embedded in the polymers will cause microorganism death over time. CA has various health benefits on human skin cells; additionally, our findings demonstrated that the cytotoxicity of CA was concentration dependent and that CA showed no cytotoxicity at low concentrations, but did have cytotoxic effects at higher concentrations. CA-loaded electrospun fibers showed relatively lower cytotoxicity compared with untreated CA. The outcome of treatment with CA may be cell- and tissue-specific. For example, although CA is known to cause allergic contact dermatitis in skin [42], it has also been reported to be immunomodulatory and reduce activation of lipopolysaccharide-stimulated macrophages [43], possibly via an anti-oxidative mechanism [44]. The cytotoxicity of CA could be explained by its effects on promoting ROS generation, reducing mitochondrial membrane potential, releasing cytochrome c, activating caspases, and inducing apoptosis in human cells [45]. However, further studies are needed to elucidate the specific mechanism.

## 5. Conclusions

In this study, PLA/CA/β-CD fibers were successfully prepared via electrospinning. Effective and proper incorporation of CA into fibers was confirmed by SEM. The CA concentration had a significant effect on the morphology of fibers. The diameter increased when the percentage of CA increased. The FTIR spectra demonstrated the presence of molecular interactions among PVA, CA, and β-CD. The mechanical properties and hydrophilicity were improved. The CA concentration in the initial fibers also influenced the CA release profile. Notably, the highest effective antibacterial activities of PLA/β-CD/CA-3 for *E. coli* and *S. aureus* were well preserved for 60 h, and cytotoxicity analysis revealed that these fibers showed no cytotoxicity toward human cells. Thus, these fibers may have applications as novel antimicrobial wound dressing materials and other biomaterials in the clinical setting.

## Figures and Tables

**Table 1:** The physical characteristics of different fibers.

Sample	Viscosity (mPa·s)	Conductivity (µS/cm)	Strain (%)	Stress (MPa)	Young’s modulus
PLA	129.15 ± 12.61 ^a^	0.037 ± 0.063 ^a^	96.74 ± 12.54 ^a^	4.42 ± 0.92 ^a^	84.62 ± 9.74 ^a^
PLA/β-CD/CA-1.5	118.46 ± 10.19 ^a^	0.039 ± 0.065 ^a^	91.25 ± 11.81 ^a^	4.31 ± 0.89 ^a^	76.47 ± 8.27 ^a^
PLA/β-CD/CA-3	97.82 ± 7.18 ^b^	0.043 ± 0.069 ^a^	88.63 ± 10.17 ^a^	4.27 ± 0.87 ^a^	73.88 ± 8.01 ^a^
PLA/β-CD/CA-6	70.48 ± 5.53 ^b^	0.049 ± 0.071 ^a^	76.36 ± 8.34 ^a^	4.02 ± 0.82 ^a^	64.21 ± 7.48 ^b^
PLA/β-CD/CA-12	45.27 ± 3.54 ^c^	0.058 ± 0.079 ^a^	70.49 ± 7.87 ^a^	3.63 ± 0.74 ^a^	52.74 ± 5.94 ^b^

Notes: ^a,b,c^ means with the same letter in the same column are not significant different (*p* > 0.05).
